# Mutant Taq DNA polymerases with improved elongation ability as a useful reagent for genetic engineering

**DOI:** 10.3389/fmicb.2014.00461

**Published:** 2014-09-03

**Authors:** Takeshi Yamagami, Sonoko Ishino, Yutaka Kawarabayasi, Yoshizumi Ishino

**Affiliations:** ^1^Protein Chemistry and Engineering, Department of Bioscience and Biotechnology, Graduate School of Bioresource and Bioenvironmental Sciences, Kyushu UniversityFukuoka, Japan; ^2^Health Research Institute, National Institute of Advanced Industrial Science and TechnologyAmagasaki, Japan

**Keywords:** thermostability, gene amplification, *in vitro* gene manipulation, *Thermus aquaticus*, PCR

## Abstract

DNA polymerases are widely used for DNA manipulation *in vitro*, including DNA cloning, sequencing, DNA labeling, mutagenesis, and other experiments. Thermostable DNA polymerases are especially useful and became quite valuable after the development of PCR technology. A DNA polymerase from *Thermus aquaticus* (Taq polymerase) is the most famous DNA polymerase as a PCR enzyme, and has been widely used all over the world. In this study, the gene fragments of the family A DNA polymerases were amplified by PCR from the DNAs from microorganisms within environmental soil samples, using a primer set for the two conserved regions. The corresponding region of the *pol* gene for Taq polymerase was substituted with the amplified gene fragments, and various chimeric DNA polymerases were prepared. Based on the properties of these chimeric enzymes and their sequences, two residues, E742 and A743, in Taq polymerase were found to be critical for its elongation ability. Taq polymerases with mutations at 742 and 743 actually showed higher DNA affinity and faster primer extension ability. These factors also affected the PCR performance of the DNA polymerase, and improved PCR results were observed with the mutant Taq polymerase.

## Introduction

In addition to their fundamental roles in maintaining genome integrity during replication and repair, DNA polymerases are widely used for genetic engineering techniques, including DNA cloning, dideoxy-sequencing, DNA labeling, mutagenesis, and other *in vitro* DNA manipulations. Among them, thermostable DNA polymerases are particularly useful for PCR and cycle-sequencing (Perler et al., 1996; Ishino and Ishino, 2013; Terpe, 2013).

The fundamental ability to synthesize a deoxyribonucleotide chain is conserved in relation to the structural conservation of the DNA polymerases. However, the more specific properties for this catalysis, including processivity, synthesis accuracy, and substrate nucleotide selectivity, differ among the enzymes. These factors should be considered when evaluating a DNA polymerase as an enzyme for genetic engineering (Ishino and Ishino, 2014). An enzyme possessing faster extension with better accuracy and higher efficiency is more preferable. In addition to these catalytic properties, thermostability is necessary for practical PCR. DNA polymerases are now classified into seven families, based on the amino acid sequences (Braithwaite and Ito, 1993; Ishino and Cann, 1998; Cann and Ishino, 1999; Ohmori et al., 2001; Lipps et al., 2003). The enzymes within the same family have basically similar properties. Commercial genetic engineering reagents have originated only from families A and B to date. The family A enzymes are used for dideoxy-sequencing, and the family A and B enzymes are used for PCR. None of the DNA polymerases from the other families are suitable for general use in genetic engineering experiments. The 3′-5′ exonuclease activity, which contributes to the proofreading of DNA strand synthesis, is generally associated with the family B enzymes, but not with the family A enzymes, although some family A enzymes have a weak 3′-5′ exonuclease activity (Joyce and Steitz, 1994; Villbrandt et al., 2000). Based on these differences, family A is advantageous for the efficient amplification of a long DNA region, and family B is generally more suitable for the precise amplification of a shorter region by PCR (Eckert and Kunkel, 1991). Researchers in this field have been making continuous efforts toward longer extension and better accuracy in PCR, and have succeeded in developing practical and reliable PCR methods. One notable example is the development of LA (long and accurate)-PCR, which is performed with a mixture of two DNA polymerases, one each from family A and family B (Barns, 1994). Further improvements of PCR have included the identification of a processive enzyme (Takagi et al., 1997) and the modifications within family B DNA polymerases that confer higher accuracy (Wang et al., 2004; Ishino et al., 2012).

Protein engineering techniques, using site-specific or random mutagenesis, are powerful ways to create mutant enzymes from the known DNA polymerases. Several useful enzymes were successfully produced by these procedures. A cold-sensitive mutant of DNA polymerase from *Thermus aquaticus* (Taq polymerase) was developed with markedly reduced activity at 37°C, as compared with the wild type (WT) enzyme (Kermekchiev et al., 2003). This mutant may be applicable to hot start PCR. Another example is a mutant Taq polymerase with enhanced resistance to various inhibitors of PCR reactions, including whole blood, plasma, hemoglobin, lactoferrin, serum IgG, soil extracts, and humic acid (Kermekchiev et al., 2009). The molecular breeding of *Thermus* DNA polymerases by a direct evolution technique (Brakmann, 2005; Henry and Romesberg, 2005; Holmberg et al., 2005; Ong et al., 2006), compartmentalized self-replication (CSR) (Ghadessy et al., 2001), also generated a PCR enzyme with striking resistance to a wide range of inhibitors (Baar et al., 2011). Furthermore, enzymes with a broad substrate specificity spectrum were also obtained by the CSR technique (Ghadessy et al., 2004; d'Abbadie et al., 2007), and are thus useful for the amplification of ancient DNA containing numerous lesions. Mutational studies in the O-helix of Taq polymerase produced enzymes with reduced fidelity (Suzuki et al., 1997, 2000; Tosaka et al., 2001), which may be useful for error-prone PCR. These studies have contributed to the elucidation of the detailed structure-function relationships of DNA polymerases, as well as to the creation of novel enzymes with different substrate specificities, stabilities, and activities from those of their naturally evolved counterparts.

In addition to the engineering of characterized enzymes to convert PCR performance, the screening for a suitable DNA polymerase activity from known organisms is the most conventional way to discover useful enzymes. However, the culturable organisms are limited, and large-scale cultivation is needed to purify an enzyme to homogeneity for precise characterization. In this study, we analyzed the DNAs from microorganisms within various soil samples obtained from a hot spring area, and compared the sequences of a region within the *pol* genes included in the environmental DNAs. We then predicted the amino acid residues that are critical for the primer extension reaction of Taq polymerase, by constructing numerous chimeric Taq polymerases including the *pol* gene fragments from the various environmental DNAs. A mutant Taq polymerase with the E742 and A743 substitutions possessed more efficient DNA strand synthesis ability and better PCR performance. This polymerase will contribute to the development of high-speed PCR with the standard PCR conditions optimized for Taq polymerase.

## Materials and methods

### Enzymes and substrates

Enzymes for *in vitro* DNA manipulation and oligonucleotides were purchased from New England Biolabs (Ipswich, MA, USA) and Sigma Aldrich (St. Louis, MO, USA), respectively. The [methyl-^3^H]TTP was purchased from Amersham (Buckinghamshire, UK) and the [γ^32^-P]ATP was purchased from NEN Life Science Products (Boston, MA, USA).

### DNA extraction

Extraction of DNA from the environmental specimens was performed with an UltraClean Soil DNA Isolation Kit (MO BIO, San Diego, CA, USA), according to the manufacturer's instructions. The extracted DNAs were assessed by agarose gel electrophoresis and were quantified by spectrophotometrical measurement.

### Construction of the expression plasmid for chimeric Taq polymerases

The expression plasmid for Taq polymerase, pTV-Taq, which contains the entire region of the structural gene encoding Taq polymerase in the pTV118N vector (Takara Bio, Shiga, Japan), was constructed exactly as described (Ishino et al., 1994). The gene in the pTV118N vector was expressed under control of the *lac* promoter and SD sequence. The pTV-Taq plasmid was subjected to site-specific mutagenesis, using QuikChange™ kit (Agilent Technologies, Santa Clara, CA, USA) to introduce BlpI (GCTNAGC) and BglII (AGATCT) restriction sites into the positions corresponding to the 5′- and 3′-termini, respectively, of the substitution region within the Taq *pol* gene. The insertion of a BglII site leads to the mutations of amino acids, Leu787Ile and Val788Leu. The resultant plasmid was named pTV-Taq', and it was used for the expression of chimeric Taq polymerases, produced by the direct substitution of the BlpI–BglII fragment with the *pol* gene fragments from the environmental DNAs, as described in detail below.

### Amplification of the *pol* gene fragments from metagenomic DNA

A region of the *pol* genes was amplified directly from the environmental DNA by PCR, using a primer set with the sequences 5′-dCGCAGGCTAAGCAGCTCC**GAYCCHAACYTSCARAAYATHCC**-3′ and 5′- dGAG**YAAGATCT**CRTCGTGNACYTG-3′, which correspond to the degenerate codons for DPNLQNIP (forward) and QVHDEIL (reverse), respectively (Y indicates C and T; H indicates A, C, and T; S indicates C and G; R indicates A and G; N indicates A, G, C, and T). The above two regions, which are conserved in the family A DNA polymerases, were successfully used to make a mixed primer set for PCR (Uemori et al., 1993). The nucleotide sequences corresponding to the conserved regions are shown in boldface, and the restriction endonuclease recognition sequences are underlined in the above primers. PCR was performed in a 50 μl reaction, containing 10 ng DNA, 25 pmol of each primer, 0.2 mM dNTP, and 1 unit of PfuUltra DNA polymerase (Stratagene). After an incubation of the mixture without the enzyme for 3 min at 95°C, thirty-cycles of PCR, with a temperature profile of 30 s at 95°C, 30 s at 55°C, and 1 min at 72°C, were performed. The reaction mixtures were electrophoresed in a 1% agarose gel, and the amplified fragments were visualized by ethidium bromide staining.

### Analysis of the amplified gene fragments

DNA fragments with a 600 bp size, amplified from the environmental DNA by PCR, were excised from the agarose gel, digested with the BlpI and BglII restriction endonucleases, and ligated into the pTV-Taq' plasmid predigested with the BlpI and BglII restriction endonucleases. The ligation mixtures were introduced into *E. coli* JM109 cells (TaKaRa Bio.), and 20 clones were picked independently from the transformants for each amplification. Plasmid DNAs were extracted from these clones, and the nucleotide sequences of the DNA inserts were determined by CEQ2000XL DNA analysis system (Beckman Coulter, USA).

### Construction of mutant Taq polymerases

The pTV-Taq plasmid was subjected to site-specific mutagenesis to introduce mutations into positions 742 and 743 of Taq polymerase. The sequences of the 14 primers used for mutagenesis are shown in Table 1. The PCR reaction mixture contained 20 ng template DNA, 1× PCR buffer for KOD-Plus-Neo, 1.5 mM Mg_2_SO_4_, 0.2 mM of each dNTP, 0.3 μM of each primer, and 0.5 unit KOD-Plus-Neo DNA polymerase (TOYOBO, Osaka, Japan) in a final volume of 20 μl. The mixture was heated at 95°C for 30 s and then subjected to thermal cycling (14 cycles of 95°C for 30 s, 55°C for 1 min, and 68°C for 8 min). The PCR product was treated with DpnI at 37°C for 1 h, and introduced into *E. coli* JM109 cells. For each mutation, the polymerase gene was fully sequenced to ensure that the mutation of interest was present and that no additional mutation was introduced by the PCR.

**Table 1:** **Oligonucletides used to introduce mutations into positions 742 and 743 of Taq polymerase**.

**Name of primer**	**Sequence (5′ to 3′)**
TaqRR-F	CGGGTGAAGAGCGTGCGCCGCCGCGCCGAGCGCATGGCC
TaqRR-R	GGCCATGCGCTCGGCGCGGCGGCGCACGCTCTTCACCCG
TaqRA-F	CGGGTGAAGAGCGTGCGCCGCGCGGCCGAGCGCATGGCC
TaqRA-R	GGCCATGCGCTCGGCCGCGCGGCGCACGCTCTTCACCCG
TaqAA-F	CGGGTGAAGAGCGTGCGCGCGGCGGCCGAGCGCATGGCC
TaqAA-R	GGCCATGCGCTCGGCCGCCGCGCGCACGCTCTTCACCCG
TaqER-F	CGGGTGAAGAGCGTGCGCGAGCGCGCCGAGCGCATGGCC
TaqER-R	GGCCATGCGCTCGGCGCGCTCGCGCACGCTCTTCACCCG
TaqAR-F	CGGGTGAAGAGCGTGCGCGCGCGCGCCGAGCGCATGGCC
TaqAR-R	GGCCATGCGCTCGGCGCGCGCGCGCACGCTCTTCACCCG
TaqRK-F	CGGGTGAAGAGCGTGCGCCGCAAAGCCGAGCGCATGGCC
TaqRK-R	GGCCATGCGCTCGGCTTTGCGGCGCACGCTCTTCACCCG
TaqKR-F	CGGGTGAAGAGCGTGCGCAAACGCGCCGAGCGCATGGCC
TaqKR-R	GGCCATGCGCTCGGCGCGTTTGCGCACGCTCTTCACCCG
TaqKK-F	CGGGTGAAGAGCGTGCGCAAAAAAGCCGAGCGCATGGCC
TaqKK-R	GGCCATGCGCTCGGCTTTTTTGCGCACGCTCTTCACCCG
TaqQY-F	CGGGTGAAGAGCGTGCGCCAGTATGCCGAGCGCATGGCC
TaqQY-R	GGCCATGCGCTCGGCATACTGGCGCACGCTCTTCACCCG
TaqAH-F	CGGGTGAAGAGCGTGCGCGCGCATGCCGAGCGCATGGCC
TaqAH-R	GGCCATGCGCTCGGCATGCGCGCGCACGCTCTTCACCCG
TaqEH-F	CGGGTGAAGAGCGTGCGCGAGCATGCCGAGCGCATGGCC
TaqEH-R	GGCCATGCGCTCGGCATGCTCGCGCACGCTCTTCACCCG
TaqHA-F	CGGGTGAAGAGCGTGCGCCATGCGGCCGAGCGCATGGCC
TaqHA-R	GGCCATGCGCTCGGCCGCATGGCGCACGCTCTTCACCCG
TaqHH-F	CGGGTGAAGAGCGTGCGCCATCATGCCGAGCGCATGGCC
TaqHH-R	GGCCATGCGCTCGGCATGATGGCGCACGCTCTTCACCCG
TaqHK-F	CGGGTGAAGAGCGTGCGCCATAAAGCCGAGCGCATGGCC
TaqHK-R	GGCCATGCGCTCGGCTTTATGGCGCACGCTCTTCACCCG

*The sites for the mutations are underlined*.

### Purification of wild type and mutant DNA polymerases

*E. coli* JM109 cells carrying the expression plasmid were grown at 37°C, in 1 L of LB medium containing 100 μg/ml ampicillin. The cells were cultured to an A_600_ of 0.2–0.3, and then the expression of the *pol* gene was induced by further cultivation for 3 h in the presence of 1 mM isopropyl-β-D-thiogalactopyranoside (IPTG). The cells were harvested and disrupted by sonication in the lysis solution (50 mM Tris-HCl, pH 8.0, 1 mM DTT, 1 mM EDTA, 1 mM PMSF). For the preparation of the WT and mutant Taq polymerases, the soluble cell extract, obtained by centrifugation at 12,000 × *g* for 20 min, was heated at 75°C for 30 min. The heat-stable fraction was obtained by centrifugation, and was treated with 0.15% (w/v) polyethyleneimine in the presence of 1 M NaCl, to remove the nucleic acids. The soluble proteins were precipitated by 80%-saturated ammonium sulfate. The precipitate was resuspended in the buffer [50 mM Tris-HCl, pH 8.0, 0.5 M (NH_4_)_2_SO_4_], and was subjected to chromatography on a hydrophobic column (HiTrap Phenyl HP 5 ml, GE Healthcare). The column was washed with 50 mM Tris-HCl, pH 8.0, and the Taq polymerase was eluted with deionized water. An equal volume of 100 mM Tris-HCl, pH 8.0, was added to this fraction, and it was then subjected to affinity chromatography (HiTrap Heparin HP 5 ml, GE Healthcare) with a gradient of 0–2 M NaCl. The proteins that eluted at 0.8 M NaCl were stored at −25°C as the final sample, in 20 mM Tris-HCl, pH 8.0, 100 mM KCl, 0.1 mM EDTA, 1 mM DTT, 0.5% NP-40, 0.5% Tween 20, and 50% (w/v) glycerol.

### Measurement of nucleotide incorporation activity

The DNA polymerizing activity was assayed by measuring the incorporation of [methyl-^3^H]TTP into the acid insoluble materials, basically as described previously (Uemori et al., 1995). To a 50 μl solution, containing 20 mM Tris-HCl, pH 8.8, 5 mM MgCl_2_, 14 mM 2-mecaptoethanol, 0.2 mM each dATP, dGTP, dCTP, and dTTP, 400 nM [methyl-^3^H]dTTP, and 20 μg of activated salmon sperm DNA, a constant amount of the enzyme fraction was added, and the reaction was incubated at 74°C for 2.5, 5, and 10 min. After the reaction, a 10 μl portion of each reaction mixture was spotted onto DE81 filters (GE Healthcare Japan, Tokyo, Japan). The filters were washed three times with a 5% Na_2_HPO_4_ solution, and the radioactivity incorporated into the DNA strands was counted by a scintillation counter. One unit of activity is defined as the amount of enzyme catalyzing the incorporation of 10 nmol of dNTP into DNA per 30 min at 74°C, and the specific activity was calculated as the units per one mg of protein (units/mg) for each DNA polymerase.

### Primer extension activity

The primer extension ability was investigated by using M13 single-stranded DNA (ssDNA) annealed with a ^32^P-labeled DNA, as described previously (Cann et al., 1999). M13 ssDNA (0.05 pmol), annealed with a 55 nucleotide long primer (5′-dTCGTAATCATGGTCATAGCTGTTTCCTGTGTGAAATTGTTATCCGCTCACAATTC-3′), was mixed with each DNA polymerase (0.05 unit) and dNTP (0.2 mM) in a 20 μl solution, containing 20 mM Tris-HCl, pH 8.8, 5 mM MgCl_2_, and 14 mM 2-mercaptoethanol, and incubated 74°C for 5 min. The reaction mixtures were analyzed by alkaline agarose gel electrophoresis, and the sizes of the products were visualized by autoradiography.

### Electrophoretic mobility-shift assay

The electrophoretic mobility-shift assay (EMSA) was performed as described previously (Komori and Ishino, 2000), to measure the DNA binding ability of the DNA polymerases. The ^32^P-labeled 27mer oligonucleotide (5′-dAGCTATGACCATGATTACGAATTGCTT-3′) was annealed with the 49mer oligonucleotide (5′-dAGCTACCATGCCTGCACGAATTAAGCAATTCGTAATCATGGTCATAGCT-3′) and was used as a DNA substrate. The radiolabeled DNA (3 nM) was mixed with DNA polymerase proteins (0.6–400 nM) in a 20 μl solution, containing 20 mM Tris-HCl, pH 8.8, 10 mM NaCl, 5 mM MgCl_2_, 14 mM 2-mercaptoethanol, 0.1 mg/ml BSA, and 5%(w/v) glycerol, and incubated at 40°C for 5 min. The DNA-enzyme mixtures were fractionated by 1% agarose gel electrophoresis. The autoradiograms were scanned and the band intensities were quantified using an image analyzer (FLA5000; Fuji Film, Tokyo, Japan). The fraction of bound DNA in each lane was calculated to be: fraction bound DNA = 1 − fraction unbound DNA. The quantitated data for binding (association) was plotted vs. enzyme concentrations. The apparent *K*d was determined to be the protein concentration at which the degree of binding equals 0.5.

## Results

### Amplification of family a DNA polymerase gene

**Figure 1:**
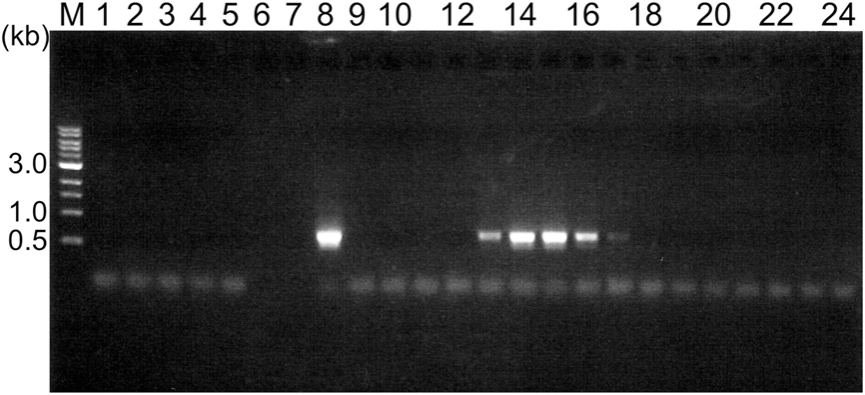
**Amplification of a region within the family A DNA polymerase gene from the environmental DNA**. PCR reaction mixtures were fractionated by 1% agarose gel electrophoresis, and the DNA bands were visualized by ethidium bromide staining. The lane numbers represent the serial numbers of the samples obtained from the hot spring areas in the Tohoku and Kyushu districts.

We collected soil samples from various hot spring areas in Japan, and isolated the DNAs within these samples. Using these DNAs as templates, a region of the *pol* genes encoding family A DNA polymerase-like sequences was amplified. We previously reported the amplification of the *pol* gene encoding a family A DNA polymerase by a set of mixed primers based on the two conserved sequence motifs (Uemori et al., 1993). The mixed primer set worked for the specific amplification of the *pol* gene fragments from the hot spring samples in this study. As shown in Figure 1 (a part of the experiments is shown), a 600 bp DNA fragment was amplified as a single band from several samples. We tested 384 different samples, and detected the amplified DNA in 37 samples, obtained at Onikobe, Hachimantai, Nasu, Kirishima, and Beppu, as shown in Table 2. These locations had various environmental conditions, including pH values of 1~7 and temperatures of mostly 70–100°C, and thus were expected to be inhabited by microorganisms with highly diverse genetic resources. The efficiency of DNA isolation was not addressed in this experiment, and thus it is possible that a sufficient amount of DNA was not present in some samples. Therefore, the result that only about 10% of the samples provided target gene amplification is not directly related to the presence or absence of microorganisms in the samples. The amplified gene fragments were excised from the gel, and were cloned into a plasmid vector. Twenty colonies were isolated independently from each cloning of the amplified DNA, and a total of 740 (20 × 37 amplifications) plasmids were isolated. These cloned DNA fragments were subjected to sequencing, and the different sequences were counted (Table 2). In total, we obtained 250 different sequences, which were not present in the public databases. This result suggested that there are still many uncharacterized DNA polymerases in the soil samples, as expected.

**Table 2:** **Summary of metagenomic analyses**.

**No**.	**Sampling place (area)**	**Temperatue (°C)**	**pH**	**Number of clones**	**Number of different clone**
1	Koyasukyo (Tohoku)	98	5	20	5
2	Koyasukyo (Tohoku)	70	7	20	12
3	Koyasukyo (Tohoku)	82	6.5	20	6
4	Koyasukyo (Tohoku)	73	–	20	16
5	Koyasukyo (Tohoku)	74	6.7	20	2
6	Koyasukyo (Tohoku)	35	–	20	10
7	Koyasukyo (Tohoku)	82	7	20	13
8	Koyasukyo (Tohoku)	50	8	20	12
9	Koyasukyo (Tohoku)	57	7	20	11
10	Koyasukyo (Tohoku)	96	4	20	4
11	Onikobe (Tohoku)	94	7	20	7
12	Onikobe (Tohoku)	95	7	20	2
13	Onikobe (Tohoku)	98	8	20	2
14	Onikobe (Tohoku)	89	7	20	9
15	Onikobe (Tohoku)	96	7	20	6
16	Onikobe (Tohoku)	98	8	20	5
17	Onikobe (Tohoku)	56	7	20	9
18	Onikobe (Tohoku)	93	1	20	6
19	Onikobe (Tohoku)	50	2	20	5
20	Onikobe (Tohoku)	85	7	20	4
21	Onikobe (Tohoku)	35	1	20	8
22	Beppu (Kyushu)	96	7	20	2
23	Beppu (Kyushu)	65	6	20	7
24	Beppu (Kyushu)	44	6	20	6
25	Beppu (Kyushu)	77	6.5	20	3
26	Beppu (Kyushu)	97	5	20	6
27	Beppu (Kyushu)	70	6	20	8
28	Beppu (Kyushu)	78	7	20	5
29	Beppu (Kyushu)	–	–	20	10
30	Kirishima (Kyushu)	75	3	20	5
31	Kirishima (Kyushu)	75	5	20	9
32	Kirishima (Kyushu)	–	5	20	6
33	Kirishima (Kyushu)	–	–	20	12
34	Ibusuki (Kyushu)	94	6	20	3
35	Nasu (Kanto)	65	6.5	20	5
36	Nasu (Kanto)	55	7	20	1
37	Nasu (Kanto)	55	6.5	20	8

“–” indicates “not analyzed.”

### Preparation of the chimeric Taq polymerases

**Figure 2:**
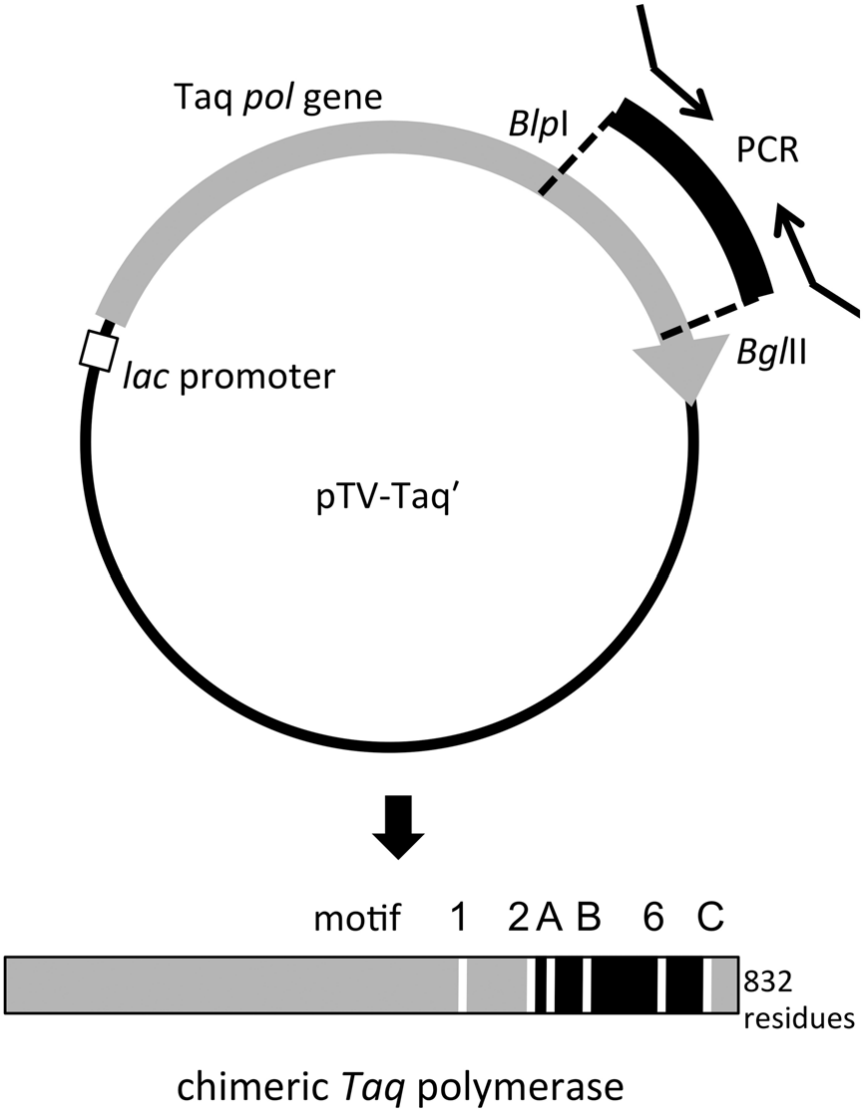
**Schematic diagram of the construction of the expression plasmid for chimeric *Taq* polymerase**. The recognition sites for BlpI (GCTNAGC) and BglII (AGATCT) were created in the Taq *pol* structural gene (gray arrow). The introduced gene fragments (black) also have the recognition sequences for these two enzymes from the PCR primers, and the substitution of the DNA fragments can be performed directly on this plasmid (upper panel). The produced chimeric protein is shown by a bar (lower panel). The replaced region was indicated in black. The motifs conserved in family A DNA polymerases are indicated by white lines (Loh and Loeb, 2005).

The amplified gene fragments encode the region in the family A DNA polymerase that is important for the nucleotide connecting reaction. To investigate the structure and function relationships of the family A DNA polymerases, we substituted the corresponding region of Taq polymerase gene with the amplified gene fragments *in vitro*. To construct the expression plasmids for the chimeric Taq polymerases systematically, restriction sites were created at appropriate sites in the Taq *pol* structural gene (Figure 2). For this purpose, the BlpI and BglII recognition sequences were suitable, although the substitutions of two amino acids, Leu797Val and Ile798Leu, could not be avoided by the introduction of BglII at the reverse priming site. The mutant Taq polymerase (L797V/I798L) was purified, and we confirmed that its fundamental properties were not affected. Therefore, the mutant Taq, designated as Taq' polymerase, was considered to be equivalent to the WT Taq polymerase. PCR primers containing the recognition sequences for BlpI (forward primer) and BglII (reverse primer) at each 5′-terminus were synthesized and each cloned DNA was re-amplified, and thus 250 chimeric genes were constructed in the Taq polymerase expression plasmid. The total cell extracts of *E. coli* producing recombinant chimeric Taq polymerases were treated at 75°C for 30 min, and the supernatants were assayed to measure the nucleotide incorporation activity. About half of the chimeric enzymes were inactivated by the heat treatment. The thermostable chimeric Taq polymerases were further purified to apparent homogeneity by the procedure described in the Materials and Methods, and the specific activity (units/mg protein) was measured by the standard DNA polymerase assay, using activated DNA. Furthermore, the primer extension ability was evaluated for each enzyme, using a constant amount (unit). These results are summarized in Table 3, which shows only the chimeric Taq polymerases possessing extension abilities better than 5 kb per 5 min-reaction. As shown in the Table 3, 13 enzymes were superior to WT Taq polymerase. However, the thermostabilities of these high-speed DNA polymerases were not sufficient for PCR applications (data not shown).

**Table 3:** **Properties of the chimeric Taq polymerase**.

**Name**	**U/mg (× 10^5^)**	**Extention kb/5 min**	***K*d for DNA (nM)**	**Sampling pace**	
				**Temperature (°C)**	**pH**
TaqWT	5.1	0.7	400	–	–
8-1	1.2	5.8	10	50	8
7-1	1.2	5.8	10	82	7
29-1	0.94	6.5	10	–	–
8-2	3.3	6.0	20	50	8
7-2	2.5	5.9	5	82	7
1-1	1.1	5.8	6	98	5
30-1	1.9	6.5	8	75	3
1-2	2.1	6.4	5	98	5
10-1	1.8	6.0	4	96	4
12-1	0.73	6.4	7	95	7
4-1	0.58	6.8	4	73	–
4-2	0.51	7.5	4	73	–
7-3	0.48	7.5	4	82	7

– indicate “not analyzed.”

### Sequence comparison of chimeric Taq polymerases and construction of the mutant Taq polymerases

**Figure 3:**
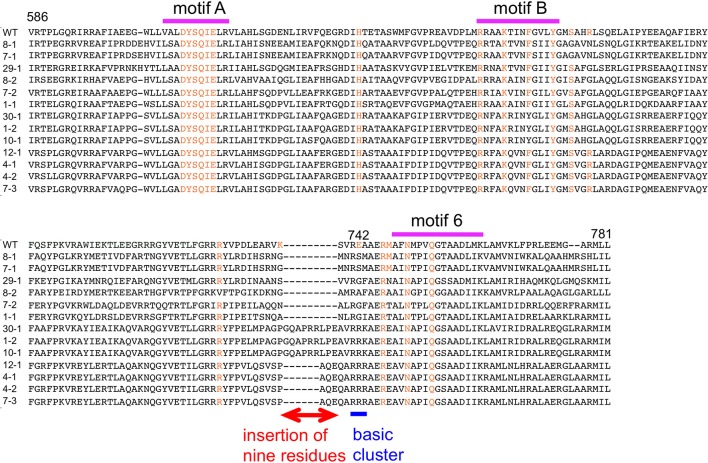
**Alignment of the amino acid sequences of chimeric Taq polymerases with extension rates over 1 kb/min**. A multiple alignment of the amino acid sequences of the substituted regions in the chimeric Taq polymerases with higher extension rates. The conserved motifs are shown on the top (Loh and Loeb, 2005). The corresponding residues involved in binding the template DNA and ddCTP, identified by the crystal structures of Klentaq (Li et al., 1998), are colored orange. The distinctive region observed in this alignment is indicated by a red line with two arrowheads. The basic cluster is indicated by a blue line.

**Figure 4:**
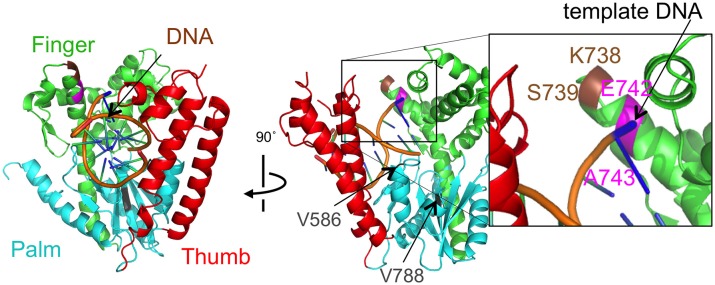
**The mutational sites of Taq DNA polymerase**. The crystal structure of Taq polymerase with DNA is shown (PDB, 1TAU). The 5′ to 3′ exonuclease domain is not illustrated for clarity. The polymerase domain is composed of a right hand with finger (green), palm (cyan), and thumb (red) subdomains (Eom et al., 1996). The thumb and finger subdomains hold DNA (backbones are colored orange). The residues E742 and A743 for the mutations are shown in magenta. The site, K738 and S739, where the interesting 9 amino acids were inserted was colored brown. The residues V586 and V788 corresponding to the junctions of the substitutions were shown in gray.

The amino acid sequences of the chimeric Taq polymerases with extension rates greater than 1 kb/min (in the condition of 0.0025 unit/μl) are aligned in Figure 3. In this alignment, we focused on the region from amino acids 730 to 745 in the Taq polymerase. One distinct feature is that 3 genes and 4 genes have insertions of 9 amino acids and 3 amino acids, respectively, as compared with WT Taq polymerase. The other characteristic feature is that continuous stretches of basic amino acids were found at residues 741–743 in the chimeric Taq polymerases. The WT Taq polymerase has Glu and Ala at 742 and 743, but many chimeric enzymes showing faster extension have Arg at both positions. The crystal structure of the large fragment of Taq polymerase (Klentaq)-DNA complex revealed that Glu742 directly interacts with the template DNA in the closed conformation, but not in the open conformation (Li et al., 1998). As shown in the right panel of Figure 4, the residues Glu742 and Ala743 (magenta) are located in the finger subdomain and face to the template DNA (blue). The basic amino acid cluster in the chimeric Taq polymerases is supposed to interact with the template DNA. We focused on this finding, and made a series of mutant polymerases by substitutions at positions 742 and 743 in WT Taq polymerase to change the affinity of the enzymes with DNA. The names of the mutant enzymes are as follows: RR (E742R/A743R), RA (E742R), AA (E742A), ER (A743R), AR (E742A/A743R), RK (E742R/A743K), KR (E742K/A743R), KK (E742K/A743K), QY (E742Q/A743Y), AH (E742A/A743H), EH (A743H), HA (E742H), HH (E742H/A743H), and HK (E742H/A743K). Fourteen mutant recombinant enzymes were purified to homogeneity from *E. coli* cells. The specific activity (units/mg protein) was measured by the standard incorporation assay (Table 4). Thermal stabilities of the mutant Taq polymerases were similar to WT enzyme (data not shown).

**Table 4:** **Properties of the mutant Taq polymerase**.

**Name**	**U/mg (× 10^5^)**	**Extention kb/5 min**	***K*d for DNA (nM)**	**Charge**
TaqEA(WT)	5.1	0.7	400	−1
TaqRR	1.3	>8	9	2
TaqRA	2.3	3.7	8	1
TaqAA	2.8	2	9	0
TaqER	1.4	3.5	8	0
TaqAR	1.1	4.3	8	1
TaqRK	1.5	>8	10	2
TaqKR	1.1	>8	10	2
TaqKK	1.4	>8	10	2
TaqQY	4.7	2.1	10	0
TaqAH	3.7	2.5	6	1
TaqEH	3.2	2.0	6	0
TaqHA	3.8	2.5	6	1
TaqHH	3.8	2.8	6	2
TaqHK	2.2	4.8	6	2

### Faster primer extension by the mutant Taq polymerases

**Figure 5:**
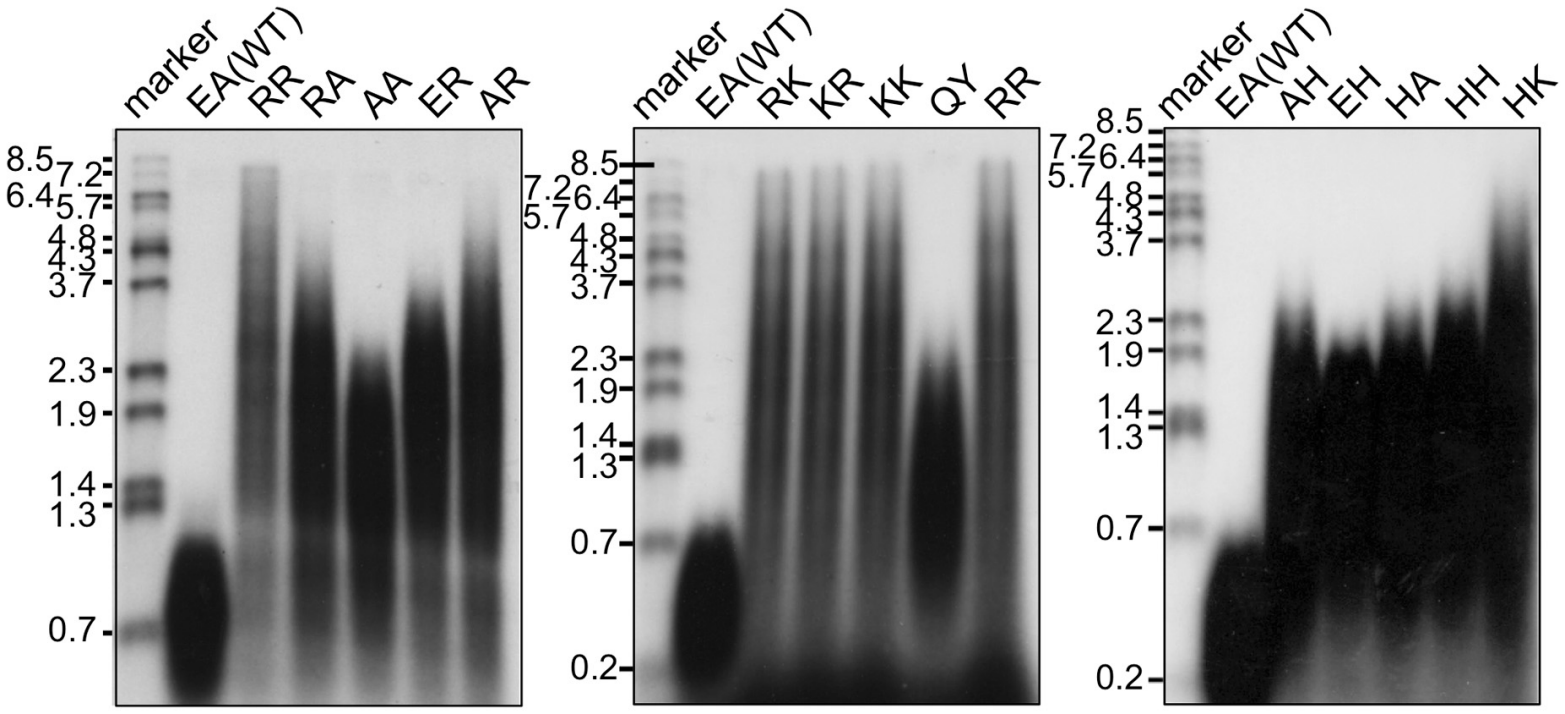
**Primer extension activities of WT and mutant Taq polymerases**. M13 ssDNA annealed with a ^32^P-labeled deoxyoligonucleotide (55mer) was used as the substrate. For each DNA polymerase, 0.05 unit was added to 20 μl reaction mixture, containing 2.5 nM primed DNA. The reaction mixtures were incubated at 74°C for 5 min, and the products were analyzed by 1% alkaline agarose gel electrophoresis, followed by autoradiography. The sizes indicated on the left are from BstPI-digested λ phage DNA labeled with ^32^P at each 5′ end. The names of the proteins were indicated on the top.

**Figure 6:**
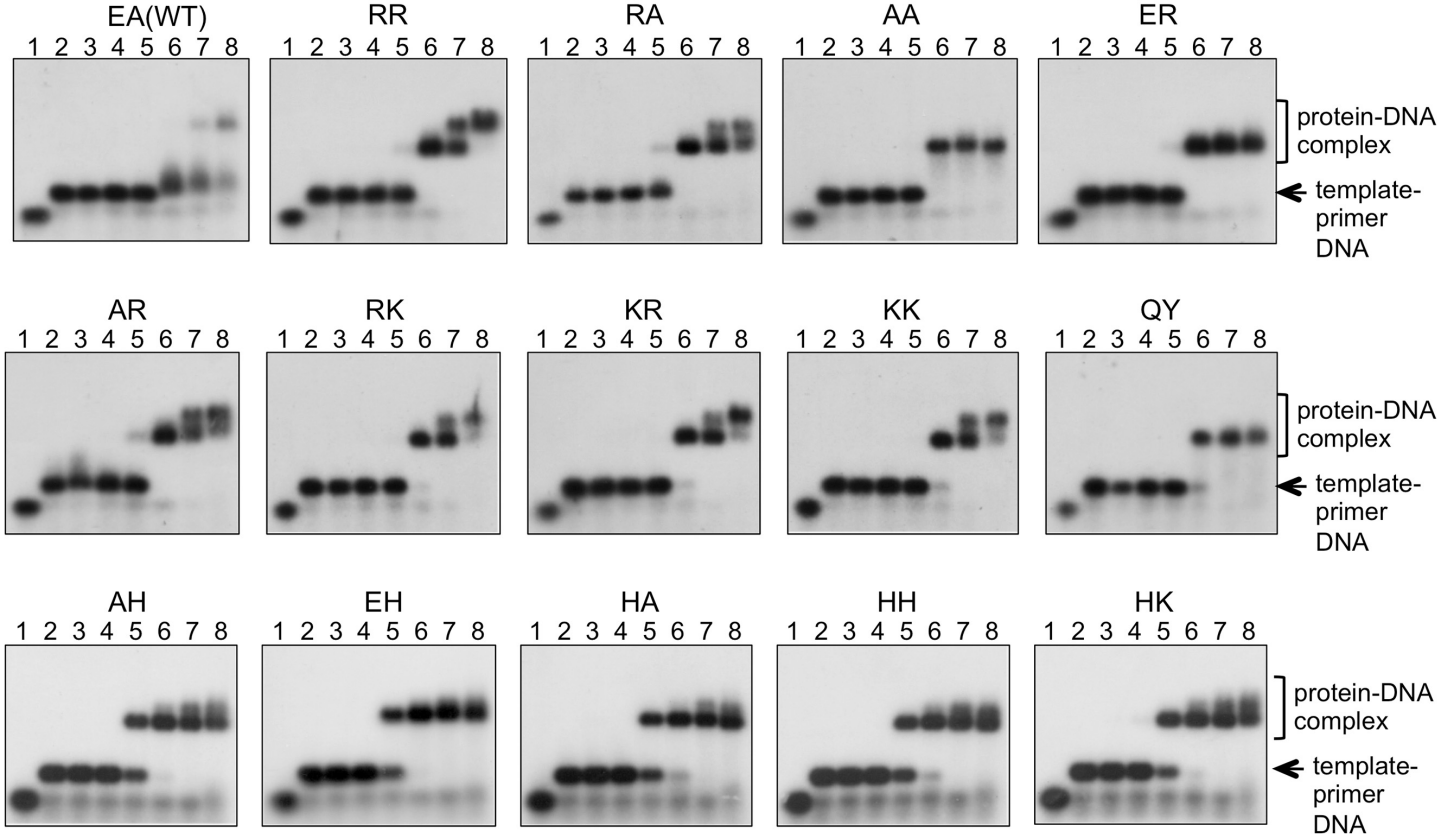
**DNA binding ability of mutant Taq polymerases**. EMSA was performed using primed DNA (^32^P-labeled 27mer DNA and 49mer DNA). The names of the mutant proteins were indicated on the top of each panel. Lane 1 was ^32^P-labeled 27mer ssDNA. Lane 2 had no protein with primed DNA. Lanes 3~8 contained 0.4, 1.6, 6.3, 25, 100, and 400 nM enzyme, respectively.

The *in vitro* primer extension rates were compared for these mutant Taq polymerases, as well as WT Taq polymerase. As shown in Figure 5, all of the mutant Taq polymerases exhibited faster extension reactions compared with that by the WT. The results of these experiments were quantified. The increased extension rate is generally related to the number of positive charges at this site (Table 4). The basic residues gave the varied effects. The positive charge of His appeared to have lower effect than those of Arg and Lys. There is a difference in pKa among the basic residues, and pKa of His, Arg and Lys are 6.8, 12.5, and 11.0, respectively. The relative degree of positive charge of His is estimated to be low. The DNA binding affinity of each enzyme was evaluated by EMSA, using a primed-DNA as a ^32^P-labeled probe. As shown in Figure 6, the DNA binding ability of all the mutant Taq polymerases was distinctly increased from that of WT Taq polymerase. The increased number of positive charge at the positions 742 and 743 appeared to provide higher binding efficiency of Taq polymerase. Apparent *K*d was determined with EMSA (Table 4). All the mutants bound to DNA by up to 2 orders of magnitude more tightly than WT Taq polymerase. Although there is no difference in the apparent *K*d among these mutants, the second-shifted bands appeared in the gel images of EMSA in the case of the mutants, which possess Arg or Lys at the positions 742 and 743. The positive charge of Arg or Lys at the positions 742 and 743 might cause a nonspecific binding, in addition to the functional binding, of the enzyme to DNA.

### Better PCR performance by the mutant Taq polymerases

**Figure 7:**
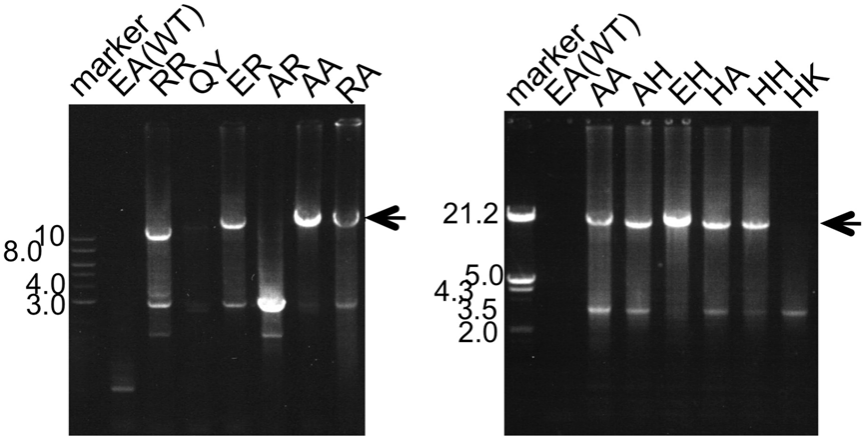
**PCR performances of WT and mutant Taq polymerases**. Lambda DNA was used as the template. Twenty nanograms of the template DNA and 5 pmol of each primer were added to the standard PCR mixture (total volume 20 μl, with 10 mM Tris-HCl, pH 8.8, 50 mM KCl, 1.5 mM MgCl_2_, 0.2 mM each dNTP and 1 unit of Taq polymerase) and 30 cycles were run (99°C for 5 s and 66°C for 5 min) in a DNA thermal cycler. A 10 μl portion of each reaction mixture was analyzed by 1% agarose gel electrophoresis. The primers designed to amplify 15 kb fragment were 15-F (5′-dGAGTTCGTGTCCGTACAACTGGCGTAATCATGGCC-3′), and 15-R (5′-dGAATATCTGGCGGTGCAATATCGGTACTGTTTGC-3′).

The main goal of this study was to create PCR enzymes with superior performance, as compared to that of WT Taq polymerase. Since the mutant Taq polymerases are as thermostable as WT enzyme, it was promising to apply these enzymes to PCR. Therefore, the PCR performances for several target DNAs with different lengths were compared. A representative example of the PCR experiments is shown in Figure 7. For the amplification of 15 kb of DNA, several mutant Taq polymerases, AA, RA, AH, EH, HA, and HH, successfully amplified the target DNA under conditions where WT Taq polymerase did not function. The other mutant enzymes prepared in this study did not work well in the same conditions. The performances of some of the mutant enzymes, RR, QY, ER, AR, and HK, are shown in Figure 7. These experiments showed inconsistency with the results of primer extension experiment. The target DNA product was not detected by the mutant enzymes that possess primer extension rate with >8 kb/5 min. The enzymes with extension rate of >8 kb/5 min have Arg or Lys at the positions 742 and 743. The observed PCR inhibition by these mutations may be due to the too tight binding to DNA, suggested by the EMSA as shown in the Section Faster Primer Extension by the Mutant Taq Polymerases. These results indicated that the positions of 742 and 743 in Taq polymerase are important for DNA strand synthesis, and the electrostatic environment of this site severely affects its PCR performance.

## Discussion

DNA polymerase is an important enzyme for both fundamental living phenomena (DNA replication/repair) in cells and applications to genetic engineering *in vitro*. Therefore, numerous structural and functional investigations of DNA polymerase have been reported to date. In this study, we developed PCR enzymes that provide a superior extension reaction as compared to Taq polymerase, the standard enzyme for PCR. As compared to the PCR performance of Taq polymerase, these enzymes achieved the amplification of either the same length of DNA in a shorter time or a longer DNA in the same reaction time.

Metagenomic analysis is a revolutionary technique for microbiological ecology. The amplification of target genes from metagenomic DNA is a very powerful method to investigate many different DNA polymerases from uncultivated microbes. In this study, we focused on thermophilic bacteria as useful genetic resources for new thermostable family A DNA polymerases. We obtained many new sequences encoding a region of a family A DNA polymerase from the hot spring soil samples. These results suggested that our strategy to amplify a specific region of the family A DNA polymerase genes is actually applicable to the analysis of microbial populations in any habitat. We employed the same strategy to search for new family B DNA polymerases, and some of this work was published previously (Matsukawa et al., 2009).

We constructed chimeric enzymes between Taq polymerase and the products of the various *pol* genes amplified from the metagenomic DNAs, and their primer extension abilities were compared. Many chimeric polymerases possessing excellent extension ability were obtained by this experiment. However, none of the chimeric enzymes were sufficiently thermostable for PCR use. The microbial sources of the gene fragments used for the construction of the chimeric genes are not necessarily extreme thermophiles, and some moderate thermophiles and mesophiles may be included among the amplified genes. The chimeric Taq polymerases showing faster extension ability than WT Taq polymerase would have gene fragments from the organisms, which are not extreme thermophiles. However, the amino acid sequence comparison of the chimeric Taq polymerases provided an important clue to design a mutant Taq polymerase with superior speed for the primer extension reaction, by site-specific mutagenesis. We focused on positions 742 and 743 in this study. The positions 742 and 743 are located in the finger subdomain and affected the interaction with DNA. The PCR performances of some of the mutant Taq polymerases showed reliable improvement, and they are useful for faster PCR and also for longer target DNAs. The conversion of the electrostatic environment at this position, from a negative charge to a positive charge, will affect the stabilization of the DNA binding near the active site of Taq polymerase. It is important to check whether the mutations in this site affect the fidelity of Taq polymerase. Our preliminary data revealed that the fidelities of these enzymes are not different from that of WT Taq polymerase (data not shown). We will confirm this with more experiments and provide statistical data in the future.

In addition to positions 742 and 743, we found one more remarkable feature in the sequences of the chimeric enzymes. These enzymes have an insertion of either 9 or 3 amino acids between positions 738 and 739 of Taq polymerase. It will be interesting to investigate the effects of these insertions in the finger subdomain on the PCR performance of Taq polymerase. Characterizations of the mutant Taq polymerases with the different inserted sequences are now underway.

In conclusion, we designed a method for engineering Taq polymerase to improve its primer extension rate, by using information obtained from the metagenomic analysis of soil samples from various hot-spring areas. The created enzymes showed robust PCR performances that were better than that of Taq polymerase. Since Taq polymerase is the standard enzyme used for PCR, an abundance of PCR data using this enzyme has been accumulated to date. The enzymes created in this study basically retain the properties of Taq polymerase, and therefore, they are applicable to many uses that have already been optimized with Taq polymerases.

### Conflict of interest statement

The authors declare that the research was conducted in the absence of any commercial or financial relationships that could be construed as a potential conflict of interest.
